# Feasibility of Routine Quality-of-Life Assessment in Long-Term Care Homes

**DOI:** 10.1093/geroni/igab046.1594

**Published:** 2021-12-17

**Authors:** Matthias Hoben, Sube Banerjee, Anna Beeber, Stephanie Chamberlain, Laura Hughes, Hannah O’Rourke, Kelli Stajduhar, Jude Spiers

**Affiliations:** 1 University of Alberta at Edmonton, Edmonton, Alberta, Canada; 2 University of Plymouth, University of Plymouth, England, United Kingdom; 3 University of North Carolina at Chapel Hill, UNC Chapel Hill, North Carolina, United States; 4 University of Alberta, University of Alberta, Alberta, Canada; 5 University of Sussex, University of Sussex, England, United Kingdom; 6 University of Victoria, University of Victoria, British Columbia, Canada; 7 University of Alberta, Edmonton, Alberta, Canada

## Abstract

Maximizing long-term care (LTC) residents' quality of life (QoL) is the primary goal of care. However, most residents have cognitive impairment and care staff time is severely limited, leading to various complexities in measuring QoL. This study developed and assessed the feasibility of an approach to routinely measuring QoL in LTC residents. We used the DEMQOL-CH, a practical, reliable, valid tool, developed in the UK to be completed by care aides to assess QoL in residents with moderate to severe dementia. We recruited 45 care aides in 10 LTC homes in Alberta, Canada who we surveyed on the QoL of 263 residents via video calls. We assessed time to complete; care aide and manager perceived feasibility of completing the DEMQOL-CH; internal consistency and inter-rater reliability of DEMQOL-CH scores; and we conducted cognitive interviews with 7 care aides to assess care aide comprehension of the tool. Time to complete was on average 4 minutes with little variation. Care aides and managers rated using the DEMQOL-CH as highly feasible and valuable. The internal consistency of the DEMQOL-CH score was 0.80. The DEMQOL-CH score inter-rater agreement was 0.73. Cognitive interviews suggested good comprehension overall with some comprehension problems especially in care aides who speak English as a second language. Asking care aides to complete the DEMQOL-CH is highly feasible, requires minor resources, and reliability is high. However, some items caused comprehension and reliability problems. Reasons and possible solutions will be subject to further investigations.

